# Hepatoprotective Potential of Partially Hydrolyzed Guar Gum against Acute Alcohol-Induced Liver Injury in Vitro and Vivo

**DOI:** 10.3390/nu11050963

**Published:** 2019-04-27

**Authors:** Chenxuan Wu, Jun Liu, Yanbin Tang, Yanxiao Li, Qiaojuan Yan, Zhengqiang Jiang

**Affiliations:** 1 Beijing Advanced Innovation Center for Food Nutrition and Human Health, China Agricultural University, Beijing 100083, China; wuchenxuan@cau.edu.cn (C.W.); junliu@cau.edu.cn (J.L.); 2 National Institute for Nutrition and Health, Chinese Center for Disease Control and Prevention, Beijing 100050, China; tangyb@ninh.chinacdc.cn; 3 Bioresource Utilization Laboratory, College of Engineering, China Agricultural University, Beijing 100083, China; liyanxiao2012@cau.edu.cn (Y.L.); yanqj@cau.edu.cn (Q.Y.)

**Keywords:** partially hydrolyzed guar gum, acute alcohol-induced liver injury, antioxidant activity, lipid metabolism, inflammation, apoptosis

## Abstract

Natural polysaccharides, particularly galactomannans, are potential candidates for treatment of alcoholic liver diseases (ALD). However, applications are restricted due to the physicochemical properties associated with the high molecular weight. In this work, guar gum galactomannans were partially hydrolyzed by β-mannanase, and the molecular mechanisms of hepatoprotective effects were elucidated both in vitro and in vivo. Release of lactate dehydrogenase and cytochrome C were attenuated by partially hydrolyzed guar gum (PHGG) in HepG2 cells, due to protected cell and mitochondrial membrane integrity. PHGG co-administration decreased serum amino transaminases and cholinesterase levels of acute alcohol intoxicated mice, while hepatic pathologic morphology was depleted. Activity of superoxide dismutase, catalase, and glutathione peroxidase was recovered to 198.2, 34.5, 236.0 U/mg protein, respectively, while malondialdehyde level was decreased by 76.3% (PHGG, 1000 mg/kg∙day). Co-administration of PHGG induced a 4.4-fold increment of p-AMPK expression, and lipid metabolism was mediated. PHGG alleviated toll-like-receptor-4-mediated inflammation via the signaling cascade of MyD88 and IκBα, decreasing cytokine production. Moreover, mediated expression of Bcl-2 and Bax was responsible for inhibited acute alcohol-induced apoptosis with suppressed cleavage of caspase 3 and PARP. Findings gained suggest that PHGG can be used as functional food supplement for the treatment of acute alcohol-induced liver injury.

## 1. Introduction

Alcoholic liver diseases (ALD), ranging from liver fibrosis to end-stage cirrhosis, account for approximately 1–4% of the total burden of diseases worldwide (World Health Organization, WHO) [1,2]. Misuse of alcohol has been considered one of the most common cause of liver diseases. Life threatening complications of portal hypertension, liver failure, and increased incidence of hepatocellular carcinoma are also related to the prevalent usage of alcohol [3]. ALD is characterized by a complex spectrum of histological lesions, such as steatosis and cirrhosis. Those basic lesions can occur separately, simultaneously, or sequentially in the same patient [2]. Various pathways have been identified to be responsible for the pathological process of ALD, such as oxidative stress, inflammation, and apoptosis [1,4].

Over consumption of alcohol can activate hepatic enzyme cytochrome P450 2E1 (CYP2E1) for alcohol metabolism [3]. Reactive oxygen species (ROS) of superoxide radicals, hydrogen peroxides, and hydroxyl radicals will be generated and accumulated in hepatocytes, inducing oxidative stresses [5]. Cellular membrane lipid components are preferential targets of ROS. Swelling and necrosis of hepatocytes will eventually be increased due to lipid peroxidation and disrupted membrane permeability [6,7]. Adenosine 5’-monophosphate (AMP)-activated protein kinase (AMPK) regulated hepatic lipid metabolism via SREBPs (sterol regulatory element binding proteins) and PPARα (peroxisome proliferator-activating receptor α) can also be affected by ROS, resulting in steatosis of hepatocytes [8]. Exposure to oxidative stress and progress of hepatic steatosis further induce liver inflammation and apoptosis [9,10]. 

Effective therapy and agents for ameliorating ALD have attracted tremendous research interest [1,4,10]. Impressive progress has been made in ALD treatment, with many drugs be developed, such as bicyclol [11], Silymarin [12], and bifendate [13]. However, there is still no satisfactory therapy for ALD treatment at present, and people are looking for alternatives [10,14]. Recently, functional ingredients, particularly polysaccharides from natural resources, have been reported to possess hepatoprotective effects [15,16]. Fucoidan, a polysaccharide extracted from *Fucus vesiculosus* can effectively decrease the level of hepatic markers in alcohol intoxicated mice [14]. Similar findings have been reported for polysaccharides from other resources, such as *Crassostrea gigas* [15], peduncles of *Hovenia dulcis* [16], *Lycium barbarum* [17], and *Hizikia fusiformis* [18].

Guar gum, the endosperm portion of *Cyamoposis tetragonolobus* L., is composed of galactomannan with β-D-(1-4)-glycosidic linked α-D-mannopyranosyl units as the backbone. Side groups of α-D-galactopyranose are attached onto the backbone at a mannose to galactose ratio (M:G) of approximately 2:1 [19]. Guar gum has been widely used in the food industry as a thickener and/or emulsion stabilizer based on its excellent solution properties [20]. However, highly viscous guar gum solution can interfere with digestion and absorption of nutrients, resulting in reduced protein efficacy and lipid utilization [21]. Moreover, extremely high viscosity restricts the incorporation of guar gum into enteral formulas or food products at a physiologically effective concentration to show positive health benefits [22]. Partially hydrolyzed guar gum (PHGG), produced by controlled enzymatic hydrolysis of guar gum, has a smaller molecular weight (MW) and is less viscous than its native form. PHGG has been primarily utilized for nutritional purposes [19,23]. Many physiological functions, including antioxidant activity [24], hypocholesterolemia and hypolipidemic effects [25], and prebiotic activity have been reported to be associated with PHGG consumption [26,27,28]. However, no report on hepatoprotective effects of PHGG has been available till now.

Antioxidant activity and regulation effects on adipose metabolism may make PHGG a potential alternative for ALD treatment. In our preliminary experiment, PHGG effectively attenuated the injury to HepG2 cells caused by alcohol intoxication. However, mechanisms underlying the hepatoprotective effects of PHGG are still poorly understood. This study was designed to evaluate the potential hepatoprotective effects of PHGG against acute alcohol-induced injury both in vitro and in vivo. Findings gained will help to expand the utilization of PHGG as a potent functional food supplement for ALD treatment.

## 2. Materials and Methods 

### 2.1. Materials

Dulbecco’s modified Eagle’s medium (DMEM), penicillin–streptomycin solution 100× (Vetec reagent grade with 10,000 units penicillin and 10 mg streptomycin/mL), and 2.5% (*w*/*w*) trypsin (2.21 mM EDTA) were provided by Corning (Manassas, VA, USA). Fetal bovine serum (FBS) was acquired from Biological Industries Inc. (Cromwell, CT, USA). EDTA-free protease inhibitor cocktail tablets and phosphatase inhibitor cocktail tablets were purchased from Roche Diagnostics Ltd. (Mannheim, Germany). Pierce’s mitochondria isolation kit was acquired from Thermo Fisher Scientific Inc. (Pittsburgh, PA, USA). Mitochondrial membrane potential detection JC-1 kit was obtained from BD Biosciences (San Jose, CA, USA). Lactic dehydrogenase (LDH) activity assay kit and bicinchoninic acid (BCA) protein assay kit were purchased from the Beyotime Institute of Biotechnology (Shanghai, China). Assay kits of superoxide dismutase (SOD), glutathione peroxidase (GSH-Px), catalase (CAT), alanine aminotransferase (ALT), aspartate aminotransferase (AST), cholinesterase (CHE), and lipid peroxidation (Malondialdehyde, MDA) were purchased from Nanjing Jiancheng Institute of Biotechnology (Nanjing, China). 

Rabbit monoclonal antibodies against Caspase-3, Cleaved Caspase-3, PARP, cytochrome C, Bcl-2, Bax, phosphor-acetyl-CoA caboxylase (p-ACC), fatty acid synthase (FASN), phosphor-adenosine 5′-monophosphate activated protein kinase (p-AMPK), beta-actin, and glyceraldehyde-3-phosphate dehydrogenase (GAPDH) were all purchased from Cell Signaling Technology Inc. (Danvers, MA, USA). Rabbit polyclonal antibody against CYP2E1 and secondary horseradish peroxidase-conjugated anti-rabbit IgG were bought from Proteintech Group, Inc (Rosemont, IL, USA). All other chemicals were of analytical grade and were used as received without further purification.

### 2.2. PHGG Preparation and Structural Features

PHGG was prepared through enzymatic hydrolysis by β-mannanase (*Rm*Man5A) from *Rhizomucor miehei* CAU432 [21]. PHGG constituents: 24.9% (*w*/*w*) manno-oligosaccharides with degree of polymerization (DP) < 7. Weight-average MW of PHGG was 2.5 × 10^4^ Da, as identified by gel permeation chromatography.

### 2.3. Cell Culture and Membrane Integrity Evaluation

Human hepatoma HepG2 cells were purchased from Peking Union Medical College Hospital (Beijing, China) and cultured in DMEM supplemented with 10% FBS and penicillin–streptomycin solution (100 U/mL penicillin and 100 μg/mL streptomycin). Cells were grown in a humidified incubator (MCO-15AC, SANYO Electric CD., Ltd., Osaka, Japan) under 5% CO_2_ atmosphere and 95% humidity at 37 °C. HepG2 cells were sub-cultured at a ratio of 1:4 every 2 days, with the culture medium being refreshed as well. 

Lactate dehydrogenase (LDH) release in alcohol intoxicated HepG2 cells was measured as indicator of hepatocytes’ membrane integrity. Briefly, HepG2 cells seeded in 96 well plates (1 × 10^4^ cells/well, 24 h) were intoxicated with 600 mM alcohol for 12 h. PHGG (0.5−2.0 mg/mL) was then added to culture medium and incubated for another 24 h. The culture medium was collected and LDH activity was quantified using a commercially available detection kit (Beyotime Institute of Biotechnology, Shanghai, China). 

### 2.4. Mitochondrial Membrane Potential Determination

Mitochondrial membrane potential (MMP) was determined using an MMP-sensitive dual-emission probe 5,5′,6,6′-Tetrachloro-1,1′,3,3′-tetraethyl-imidacarbocyanine iodide (JC-1). After alcohol intoxication, HepG2 cells were stained with JC-1 working solution at 37 °C for 20 min. Stained cells were then washed three times with PBS and subjected to flow cytometric analysis (Becton Dickinson FACS Calibur flow cytometer, BD Biosciences, San Jose, CA, USA). The fluorescence of JC-1 aggregates (red color) formed in intact mitochondria and monomers (green color) formed in depolarized mitochondria was measured using filter pairs of 530 nm/590 nm and 485 nm/538 nm, respectively. Ratio of fluorescence of aggregates to monomers was used as the indicator of MMP. Data were analyzed with CellQuest software (BD Biosciences, San Jose, CA, USA) and normalized to control cells.

### 2.5. Preparation of Mitochondria

Mitochondria were isolated from cytosolic components using Pierce’s mitochondria isolation kit. Briefly, cell suspension was homogenized with a Dounce homogenizer (Kontes, Vinelsnd, NJ, USA) on ice and centrifuged at 1000×*g* and 4.0 °C for 3 min. Cell pellets were collected and re-suspended in mitochondria isolation reagent based on the manufacturer’s instructions. The suspension was centrifuged at 700×*g* and 4.0 °C for 10 min. Supernatant was collected and again centrifuged at 3000× *g* and 4.0 °C for 15 min. Mitochondrial pellet was collected and lysed in RIPA lysis buffer, and stored frozen until subsequent analysis.

### 2.6. Animals and Grouping

Healthy female Kunming mice (21–25 g, 4 weeks of age) were obtained from the National Institutes for Food and Drug Control (Beijing, China). All experiments were performed under standard laboratory conditions of temperature (25 ± 2 °C) and relative humidity (55 ± 5%) with a 12 h light/dark cycle. Mice were housed in polypropylene cages (29 × 18 × 16 cm), with free access to a basal diet (Vital River Laboratory Animal Technology Co., Ltd., Beijing, China) and water. Maintenance and treatment of all animals were in compliance with the principles of the Institutional Animal Ethics Committee of the Chinese Center for Disease Control and Prevention, and conformed to the Chinese national guidelines on the care and use of laboratory animals. 

Mice were randomly divided into six experimental groups (*n* = 10/group): (1) Control group, treated with saline only; (2) Alcohol group, treated with saline plus alcohol; (3) Bifendate + Alcohol group, treated with 150 mg/kg∙day bifendate plus alcohol; (4) PHGG high dose + alcohol group (PHGG-H + Alcohol), treated with 2000 mg/kg∙day of PHGG plus alcohol; (5) PHGG medium dose + alcohol group (PHGG-M + Alcohol), treated with 1000 mg/kg∙day of PHGG plus alcohol; (6) PHGG low dose + alcohol group (PHGG-L + Alcohol), treated with 500 mg/kg∙day of PHGG plus alcohol. Body weight and food consumption of the mice were monitored once a week. No adverse effects were noted regarding behavior and general health of animals exposed to PHGG. The healthy female Kunming mice in each experimental group orally received corresponding test samples stated above by gavage once a day for four weeks. Two hours after the last administration, ethanol (50%, *v*/*v*) was intragastrically administrated once at a dosage of 14 mL/kg. At the end of experiment period, all mice were fasted for 16 h and blood samples were collected from orbit. Subsequently, the mice were sacrificed and liver tissues were excised immediately. 

The protocol was approved by the Laboratory Animal Welfare & Ethics Committee (LAWEC), National Institute for Nutrition and Health, China (NHPF06-41-01).

### 2.7. Serum Biochemical Analysis

Blood samples were kept at 4 °C for coagulation (4 h). Serum was separated through centrifugation at 3500 rpm and 4 °C for 10 min. Levels of ALT, AST, and CHE were determined using commercially available diagnostic kits (Nanjing Jiancheng Bio Co., Nanjing, China). Tumor necrosis factor (TNF)-α, interleukin (IL)-6, IL-1β, and adiponectin (ADPN) in blood serum were measured by corresponding ELISA kits (Biolegend, San Diego, CA, USA; XinleBio, Shanghai, China). Triglyceride (TG), total cholesterol (TC), free fatty acids (FFA), and low density lipoprotein-cholesterol (LDL-C) were determined using a Mindray BS-420 Automatic Analyzer (Mindray, Shenzhen, China). 

### 2.8. Lipid Peroxidation and Antioxidant Enzyme Activities

Liver tissues were homogenized on ice with 1×PBS (pH 7.4, 10% *w*/*v*), then centrifuged at 3000 rpm for 20 min at 4 °C. Supernatants were used to measure activities of antioxidant enzymes SOD, GSH, GSH-Px, and CAT, as well as MDA content, employing commercially available diagnostic kits (Nanjing Jiancheng Bio Co., Nanjing, China).

### 2.9. RNA Isolation and Gene Expression Quantification

Total RNA was isolated from liver tissues with Trizol Reagents (Life technologies, Carisbad, CA, USA). The RNA was reverse-transcribed into complementary (c) DNA using a high-capacity cDNA reverse transcription kit (Promega Biotech Co., Ltd., Madison, WI, USA). Genes of PPARα, SREBP1, toll like receptor (TLR)-4, MyD88, IκBα, and NOS2 were amplified through real time-quantitative polymerase chain reaction (RT-qPCR) with the primers shown in Table 1. GAPDH was used as internal control in all reactions. A LightCycler^®^ 96 RT-qPCR system (Roche, Mannheim, Germany) was employed, with 20 μL reaction volume containing 12.5 μL POWER SYBR green master mix (TAKARA, Dalian, China), 5 μmol/L primer, and 25 ng cDNA template. Reaction was performed with an initial hold step at 95 °C for 30 s, followed by 40 cycles of 95 °C for 5 s, 60 °C for 30 s, and 72 °C for 30 s. Melting curves of PCR products were acquired stepwise from 55 to 95 °C to ensure their purity. Comparative CT method was employed for target gene quantification, and to be normalized to GAPDH and relative to a calibrator (2^−ΔΔCt^). 

### 2.10. Western Blot Analysis

Treated HepG2 cells or frozen liver tissues were lysed with RIPA lysis buffer (Midi) supplemented with a 1/200 dilution of protease and phosphatase inhibitor cocktails (Roche Diagnostics Ltd., Mannheim, Germany) for 30 min. Proteins in lysates were collected through centrifugation (12,000× *g*, 4 °C, 15 min) and determined by BCA method. Sodium dodecyl sulfate-polyacrylamide gel electrophoresis was employed for protein (50 μg) separation and electro-transferred onto a nitrocellulose filter membrane (Cell Signaling Technology Inc., Danvers, MA, USA). Membranes were blocked for 2 h with 5% nonfat milk solution in Tris-buffered saline containing 0.1% Tween 20. Blocked membranes were then incubated overnight (4 °C) with specific primary antibodies against CYP2E1 (1:1000 dilution), p-AMPK (1:1000 dilution), ACC (1:1000 dilution), FASN (1:1000 dilution), Bcl-2 (1:1000 dilution), Bax (1:1000 dilution), Caspase 3 (1:1000 dilution), cleaved Caspase 3 (1:1000 dilution), PARP (1:1000 dilution), cytochrome C (1:1000 dilution), GAPDH (1:2000 dilution), and β-actin (1:2000 dilution). Immunoblots were washed three times with Tris-buffered saline containing 0.1% Tween 20 (each for 10 min), followed by a secondary horseradish-peroxidase-conjugated anti-rabbit IgG at room temperature for 1 h. Proteins were detected with chemiluminescent ECL assay kit (Bio-Rad, Hercules, CA, USA) following manufacturer’s instructions. Optical density of each protein band was measured by ImageJ software (National Institutes of Health, Bethesda, MD, USA).

### 2.11. Histopathological and Apoptosis Analysis

Fresh liver tissues from the edge of left lobe (0.5 cm) were fixed immediately with 10% formaldehyde. After routine dehydration, transparency, and paraffin embedding, tissues were sliced to 5 μm thick and stained with hematoxylin and eosin (H&E). Histomorphology examination was conducted under a light microscope (Olympus BX60; Olympus Corporation, Tokyo, Japan). 

Apoptosis was analyzed by terminal deoxynucleotidyl transferase mediated d-UTP nick end labeling (TUNEL) staining based on the manufacturer’s instructions (Boster Biological Technology, Wuhan, China). Paraffin-embedded tissue sections were incubated with TUNEL reaction mixture at 37 °C for 120 min, followed by blocking reagent at room temperature for 30 min. Tissue sections were then reacted with anti-DIG-Biotin in a humidified chamber for 30 min (37 °C). Finally, DNA fragmentation that happened in the course of apoptosis of liver cells was visualized by SABC-FITC treatment.

### 2.12. Statistical Analysis

Data are presented as the mean ± standard deviation (SD) of three independent experiments. Statistical significance of difference was determined using one-way analysis of variance, followed by multiple comparisons with Dunnett’s test. *p* < 0.05 was considered statistically significant.

## 3. Results

### 3.1. Attenuation of Alcohol-Induced Cytotoxicity by PHGG in Vitro

**Figure 1:**
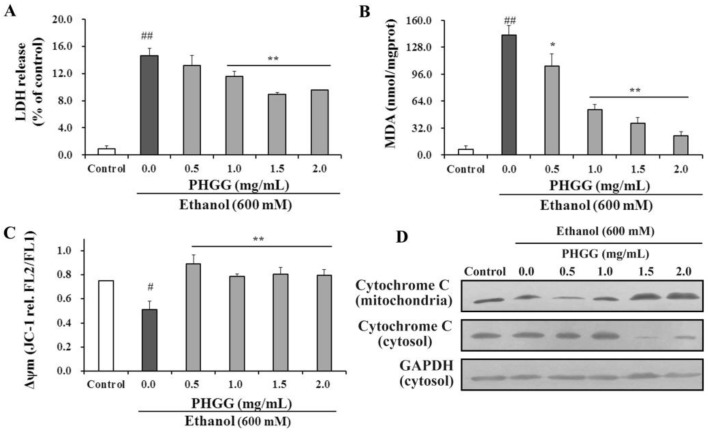
Effect of partially hydrolyzed guar gum (PHGG) on alcohol-induced cytotoxicity. HepG2 cells were intoxicated with 600 mM alcohol for 12 h prior to PHGG protection (0.5–2.0 mg/mL, 24 h). (**A**) Release of lactic dehydrogenase (LDH) was determined and used as an indicator of cell membrane integrity. (**B**) Malondialdehyde (MDA) levels in cells were determined and used as an indicator of cell lipid peroxidation. (**C**) Mitochondrial membrane potential (MMP) of alcohol intoxicated HepG2 cells with/without PHGG protection was determined by JC-1 assay. Ratio of fluorescence of aggregates (FL2) to monomers (FL1) was used as the indicator of MMP. (**D**) Western blot images of cytochrome C release from mitochondria to cytosol in alcohol intoxicated HepG2 cells treated with PHGG for 24 h. Results shown here are mean ± SD of three independent experiments. “#” (*p* < 0.05) and “##” (*p* < 0.01), significantly different when compared to the control group. “*” (*p* < 0.05) and “**” (*p* < 0.01), significantly different when compared to the alcohol group.

LDH release is commonly used as indicator of hepatocytes membrane integrity and organelle damage. As shown in Figure 1A, alcohol treatment significantly increased LDH release from HepG2 cells. In the presence of PHGG, cytotoxicity of alcohol was attenuated, with LDH release suppressed in a dose dependent manner. At PHGG concentration of 1.5 mg/mL, LDH release was 8.61%, which was significantly lower than that of untreated cells (14.95%). MDA formation in alcohol intoxicated HepG2 cells was also significantly suppressed by PHGG treatment in a dose dependent manner (Figure 1B). In the absence of PHGG, alcohol intoxication significantly increased MDA formation (142.4 nmol/mg protein) as compared with the control (6.67 nmol/mg protein). PHGG incubation efficiently attenuated alcohol-induced MDA formation, with 83.7% of MDA equivalent cell lipid peroxidation suppressed at PHGG 2.0 mg/mL.

As shown in Figure 1C, alcohol intoxication of HepG2 cells induced a significant decrease of mitochondrial membrane potential (Δψm = 0.75) as compared with untreated cells (Δψm = 0.51). Due to the altered permeability of cell membrane, release of cytochrome C from mitochondria to the cytosol was increased, as indicated in Figure 1D. However, PHGG protected cell membrane integrity at concentrations higher than 0.5 mg/mL (Figure 1C). Accordingly, release of cytochrome C was suppressed by PHGG treatment in a dose dependent manner (Figure 1D). 

### 3.2. Protection against Alcohol-Induced Liver Injury by PHGG in Vivo

**Figure 2:**
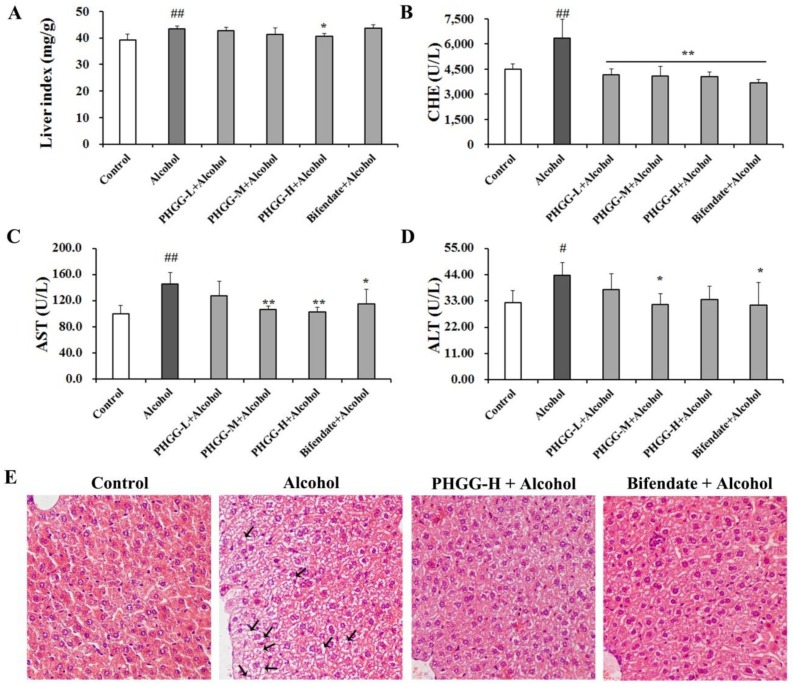
Effect of partially hydrolyzed guar gum (PHGG) on alcohol-induced liver damage. Liver index (**A**), serum cholinesterase (CHE) (**B**), aspartate aminotransferase (AST) (**C**), and alanine aminotransferase (ALT) (**D**) levels were measured as indicators of alcohol-induced liver damage. Histomorphology examination from mice of different treatment groups—Control group, Alcohol group, PHGG-H + Alcohol group, and Bifendate (150 mg/kg∙day) + Alcohol group on liver sections (1 : 200)—was visualized by H&E staining (**E**). Results are shown as mean ± SD (*n* = 10) of three independent experiments. “#” (*p* < 0.05) and “##” (*p* < 0.01), significantly different when compared to the Control group. “*” (*p* < 0.05) and “**” (*p* < 0.01), significantly different when compared to the Alcohol group. PHGG-L + Alcohol: PHGG low dose + alcohol group, treated with 500 mg/kg∙day of PHGG plus alcohol; PHGG-M + Alcohol: PHGG medium dose + alcohol group, treated with 1000 mg/kg∙day of PHGG plus alcohol; PHGG-H + Alcohol: PHGG high dose + alcohol group, treated with 2000 mg/kg∙day of PHGG plus alcohol.

Body weight gain and liver index are used as indicators of alcoholic toxicity. When compared with the Control group, mice in the Alcohol group showed significantly decreased body weight. However, co-administration of high dose PHGG (2000 mg/kg∙day) with alcohol significantly inhibited mice body weight reduction as compared with the Alcohol group. Consistently, alcohol consumption significantly increased the liver index of mice as compared with that of Control group. The increased liver index caused by alcohol administration was diminished with high dose PHGG supplementation in the diet (2000 mg/kg∙day) (Figure 2A). 

Serum amino transaminases (ALT and AST) and cholinesterase (CHE) are specific biomarkers for hepatocellular damage. As shown in Figure 2B–D, serum levels of ALT, AST, and CHE were significantly increased after alcohol administration as compared with the control group. PHGG co-administration with alcohol significantly alleviated alcoholic liver damage, with significantly decreased serum ALT, AST, and CHE levels. At a PHGG dosage of 1000 mg/kg∙day, serum levels of ALT (37.5 U/L), AST (127.8 U/L), and CHE (4157.4 U/L) were comparable with those of the Bifendate + Alcohol group. 

Pathologic morphology of liver tissues was investigated by H&E staining. Significant differences of liver section structure were observed among Control group, Alcohol group, Bifendate + Alcohol group, and PHGG-H (2000 mg/kg∙day) + Alcohol group (Figure 2E). Healthy morphology of clear and integrity was observed in the Control group, with hepatic cells arranged regularly. However, after alcohol administration, cell arrangement was disordered and ill-defined hepatocyte boundaries, vacuolated cytoplasm, and hepatic steatosis appeared, as indicated by the arrows. Supplementation of PHGG or bifendate depleted alcohol-induced hepatic pathological changes, with liver cells basically arranged regularly. 

MDA level and activities of cellular antioxidant enzymes (SOD, CAT, and GSH-Px) in liver tissues are shown in Table 2. As compared with the Control group, the SOD, CAT, and GSH-Px activity of the Alcohol group were significantly decreased to 154.2, 21.4, and 143.8 U/mg protein, respectively. However, the MDA level in Alcohol group (6.64 nmol/mg protein) was significantly higher than that of th eControl group (1.56 nmol/mg protein). PHGG supplementation significantly recovered the activities of cellular antioxidant enzymes, while MDA formation caused by lipid peroxidation in alcohol exposed groups was suppressed. At a PHGG dosage of 1000 mg/kg∙day, CAT and GSH-Px activity were 34.7 and 236.1 U/mg protein, respectively, which was comparable with that of the Control group. SOD activity in the PHGG-M (1000 mg/kg∙day) + Alcohol group was 198.8 U/mg protein, which was not significantly different to that of the Bifendate + Alcohol group (194.4 U/mg protein). Lipid peroxide formation was decreased by 76.3% of MDA equivalent level (1.57 nmol/mg) after co-administration of PHGG (1000 mg/kg∙day) with alcohol.

### 3.3. Regulation on Lipid Metabolism by PHGG in Alcoholic Fatty Liver 

The effects of PHGG administration on serum lipids of LDL-C, TC, TG, and FFA, were investigated. As shown in Table 3, alcohol induced a significant increase of serum LDL-C (0.95 mmol/L), TC (4.01 mmol/L), TG (2.03 mmol/L), and FFA (0.48 mmol/L) as compared with the Control group. Co-administration of PHGG (1000 mg/kg∙day) significantly reduced LDL-C, TC, TG, and FFA content to 0.67, 2.69, 2.11, and 0.31 mmol/L, respectively, which was comparable with that of Bifendate + Alcohol group. For the PHGG-M (1000 mg/kg∙day) + Alcohol group, serum ADPN level (10.61 mg/L) was recovered to the positive control level (11.30 mg/L), which was significant higher than that of the Alcohol group (6.99 mg/L). 

**Figure 3:**
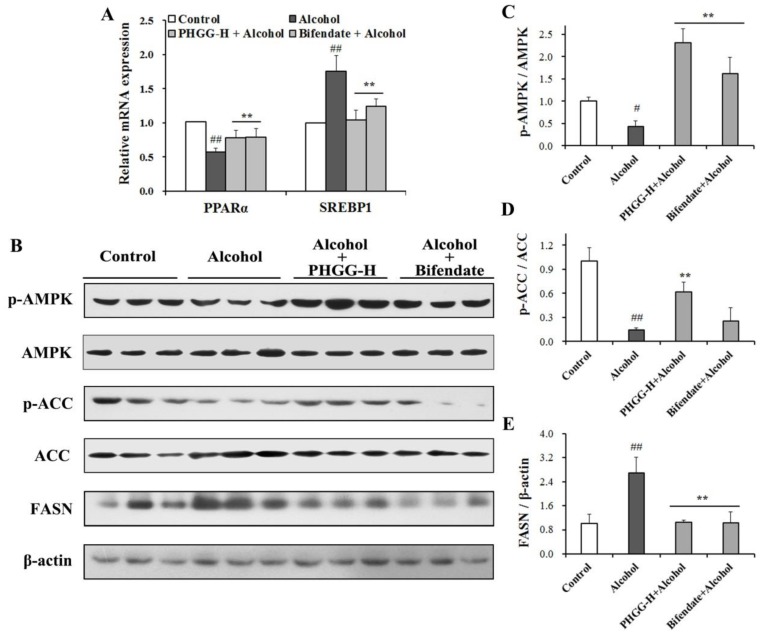
Effect of partially hydrolyzed guar gum (PHGG) on alcohol-induced liver damage. (**A**) Gene expressions of PPARα and SREBP1 in liver tissues were quantified by RT-qPCR analysis. (**B**) Western blot images of p-AMPK, p-ACC, and FASN. Densitometric blots of p-AMPK (**C**), p-ACC (**D**), and FASN (**E**) were produced by measuring protein band density using ImageJ software. Liver tissues were collected from mice of different treatment groups: control group, alcohol group, PHGG-H + alcohol group, and bifendate + alcohol group. Results are shown as mean ± SD (*n* = 3) of three independent experiments. “#” (*p* < 0.05) and “##” (*p* < 0.01), significantly different when compared to the control group. “**” (*p* < 0.01), significantly different when compared to the alcohol group. PHGG-H + Alcohol: PHGG high dose + alcohol group, treated with 2000 mg/kg∙day of PHGG plus alcohol.

SREBP1 and PPARα are responsible for hepatic lipid metabolism. The qPCR results (Figure 3A) demonstrated that PPARα expression was significantly decreased by alcohol administration by 44.0% when compared with the control group. Co-administration of PHGG markedly kept PPARα at normal level in alcoholic liver tissues. Conversely, SREBP1 expression in liver tissues was up-regulated by 74.9% after alcohol administration. Both PHGG and bifendate supplementation significantly decreased the activation of SREBP1 induced by alcohol. AMPK is a multi-subunit protein kinase that acts as a key metabolic “master switch” in lipid metabolism [8]. Alcohol exposure suppressed 57.5% p-AMPK expression as compared with the control group, while co-administration of PHGG induced 4.4-fold increase of p-AMPK (Figure 3B,C). Consistently, p-ACC for fatty acid oxidation restraining was down-regulated by 85.9% after alcohol treatment (Figure 3B,D). Meanwhile, expression of FASN for catalyzing acetylcoa and malonyl coenzyme A to produce fatty acids was up-regulated by 1.7-fold as compared with the Control group (Figure 3B,E). PHGG supplementation for 4 weeks recovered the expressions of p-ACC and FASN in the acute alcohol intoxicated liver tissues (Figure 3D,E). 

### 3.4. Alleviation of TLR4 Mediated Inflammatory Responses by PHGG

**Figure 4:**
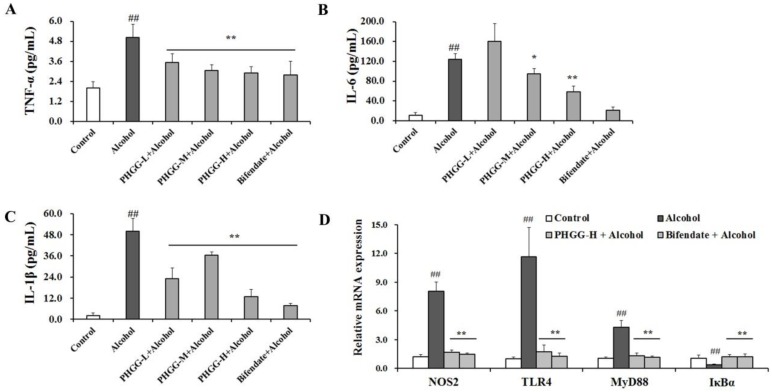
Effect of partially hydrolyzed guar gum (PHGG) on alcohol-induced inflammatory responses. Serum levels of tumor necrosis factor (TNF)-α (**A**), interleukin (IL)-6 (**B**), and IL-1β (**C**) in alcohol intoxicated mice with or without PHGG protection were determined and used as indicators of inflammatory responses. Results are shown as mean ± SD (*n* = 10). (**D**) Gene expressions of NOS2, toll like receptor (TLR)-4, MyD88, and IκBα in liver tissues from mice of the Control group, Alcohol group, PHGG-H + Alcohol group, and Bifendate + Alcohol group were quantified by RT-qPCR analysis. Data are expressed as mean ± SD (*n* = 5) of three independent experiments. “##” (*p* < 0.01), significantly different when compared to the control group. “*” (*p* < 0.05) and “**” (*p* < 0.01), significantly different when compared to the alcohol group. PHGG-L + Alcohol: PHGG low dose + alcohol group, treated with 500 mg/kg∙day of PHGG plus alcohol; PHGG-M + Alcohol: PHGG medium dose + alcohol group, treated with 1000 mg/kg∙day of PHGG plus alcohol; PHGG-H + Alcohol: PHGG high dose + alcohol group, treated with 2000 mg/kg∙day of PHGG plus alcohol.

As shown in Figure 4, alcohol consumption triggered inflammatory responses, with serum levels of IL-6, IL-1β, and TNF-α significantly elevated as compared with normal control group. Bifendate supplementation recovered the cytokines to levels comparable to the Control group. High-dosage PHGG (2000 mg/kg∙day) significantly reduced the release of IL-1β, IL-6, and TNF-α by 74.4%, 52.9%, and 41.2% after four weeks of consumption (Figure 4A–C). Furthermore, underlying mechanisms involved in the protective effect of PHGG against acute alcohol-induced inflammatory responses were investigated. Expression of genes NOS2, TLR4, MyD88, and IκBα was quantified by RT-qPCR (Figure 4D). In the alcohol intoxicated group, NOS2, TLR4, and MyD88 expression levels were markedly increased when compared with those of Control group. After supplementation with PHGG (2000 mg/kg∙day) for four weeks, expression levels of NOS2, TLR4, and MyD88 were decreased by 79.2%, 85.0%, and 69.3%, respectively, and were comparable with those of the Bifendate + Alcohol group. However, PHGG (2000 mg/kg∙day) significantly alleviated the alcohol consumption induced suppression of IκBα hepatic expression. Supplementation of PHGG or bifendate maintained IκBα gene expression at a normal level (Figure 4D).

### 3.5. Suppression of Alcohol-Induced Hepatocyte Apoptosis by PHGG

**Figure 5:**
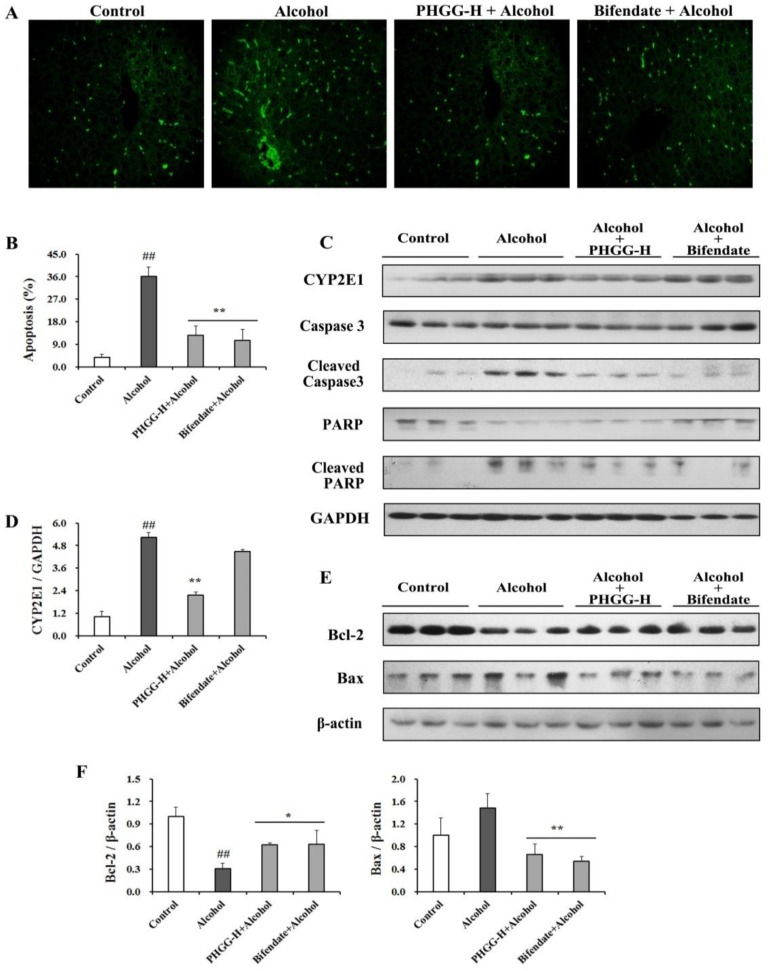
Effect of partially hydrolyzed guar gum (PHGG) on alcohol-induced apoptosis in liver tissues. (**A**) Terminal deoxynucleotidyl transferase mediated d-UTP nick end labeling (TUNEL) staining of liver tissues from alcohol intoxicated mice with/without PHGG protection. (**B**) Apoptosis of liver tissues from alcohol intoxicated mice with/without PHGG protection. (**C**) Western blot images of cytochrome P450 2E1 (CYP2E1), Caspase 3, and PARP in ALD mouse with/without PHGG protection. (**E**) Western blot images of Bcl-2 and Bax protein expression in liver tissues from alcohol intoxicated mice with/without PHGG protection. Beta-actin and GAPDH were used as loading control. Densitometric blots of CYP2E1 (**D**), Bcl-2, and Bax (**F**) were produced by measuring protein band density using ImageJ software. Liver tissues were collected from mice of the Control group, Alcohol group, PHGG-H + Alcohol group, and Bifendate + Alcohol group. Results are shown as mean ± SD (*n* = 3) of three independent experiments. “##” (*p* < 0.01), significantly different when compared to the Control group. “*” (*p* < 0.05) and “**” (*p* < 0.01), significantly different when compared to the Alcohol group. PHGG-H + Alcohol: PHGG high dose + alcohol group, treated with 2000 mg/kg∙day of PHGG plus alcohol.

Cell apoptosis in liver tissues was examined by terminal deoxynucleotidyl transferase mediated d-UTP nick end labeling (TUNEL) assay. Large apoptotic cells with high intensity green fluorescence were observed in liver tissues from alcohol exposed mice (Figure 5A). Both PHGG and bifendate significantly suppressed cell apoptosis induced by alcohol administration, as indicated by decreased green fluorescence intensity. The cell apoptosis rate in liver tissues from the Bifendate + Alcohol group (150 mg/kg∙day) and the PHGG-H (2000 mg/kg∙day) + Alcohol group was consistently decreased by 10.6% and 12.5%, respectively, when compared with the Control group (Figure 5B). Expression of CYP2E1 proteins in the Alcohol groups with or without PHGG supplementation was examined by Western blot assay (Figure 5C). Compared with the Control group, alcohol significantly improved CYP2E1 protein expression. However, alcohol-induced CYP2E1 protein expression was significantly attenuated after four weeks of PHGG supplementation (2000 mg/kg∙day) (Figure 5D).

For apoptosis induced by alcohol treatment, cleavage of both Caspase 3 and PARP was significantly increased, as indicated in Figure 5C. Both PHGG (2000 mg/kg∙day) and bifendate (150 mg/kg∙day) significantly reduced cleavage of Caspase 3 and PARP induced by alcohol consumption. Regulation effect of PHGG on Bcl-2 family proteins expression was analyzed. Compared with the Control group, expression of pro-apoptotic protein Bax was stimulated while anti-apoptotic protein Bcl-2 expression was inhibited after alcohol consumption. PHGG supplementation increased expression level of Bcl-2 1.1-fold, while expression of Bax was suppressed by 53.8% (Figure 5E,F). Both Bcl-2 and Bax expressions in Alcohol group were recovered to Bifendate + Alcohol group comparable levels after four weeks of PHGG administration (2000 mg/kg∙day) (Figure 5F).

## 4. Discussion

The prevalence of ALD worldwide requires effective therapies and agents for treatment. The antioxidant activity and adipose metabolism regulative effects of PHGG may enable it as a potential alternative of therapy for acute ethanol-induced liver damage [24,25]. In this work, the hepatoprotective effects of PHGG against alcohol-induced liver injuries were studied for the first time. PHGG can effectively attenuate acute alcohol-induced liver injury both in vitro and in vivo. Different metabolic pathways, such as antioxidant activities, lipid peroxidation reduction, suppression of inflammatory mediator expression, and/or anti-apoptosis activities, possibly contribute to the hepatoprotective effects of PHGG. Oxidative stress, the imbalance of prooxidants and antioxidants, has been considered as a putative mechanism underlying ALD [5]. Alcohol metabolism in liver tissues mediates oxidative stress through the generation of ROS [9]. Consequently, hepatocyte biochemistry will be disturbed with membrane peroxidation or other cell injuries [7]. In this study, PHGG prevented alcohol-induced peroxidation of HepG2 cell membrane lipids, and maintained membrane integrity and mitochondrial membrane structure, thus reducing the alcohol-induced release of LDH and apoptotic cytochrome C (Figure 1). Those are consistent with the previous findings that PHGG could act as an effective scavenger against superoxide and free radicals [24,29].

Serum activities of CHE, AST, and ALT are sensitive liver function indexes and are usually employed for the diagnosis of liver diseases [10]. When serious liver damage occurs, these enzymes are leaked out of the cytochylema into the blood. In the present study, PHGG supplementation significantly decreased serum CHE (Figure 2B), AST (Figure 2C), and ALT (Figure 2D) levels of the Alcohol group. AST and ALT levels of ALD Kunming mice were effectively alleviated by the polysaccharide from *maca*, while excessive consumption increased the metabolism burdens in the liver [30]. Histopathological examination on H&E-stained liver tissue slices further confirmed the hepatoprotective effects of PHGG (Figure 2E). Disordered cell arrangement, ill-defined cell morphology, edema, and slight steatosis induced by acute alcohol administration were markedly recovered by PHGG supplementation. *Auricularia cornea* var. *Li*. polysaccharides (APS) were reported to protect liver tissue against acute alcohol toxicity. Cellular architecture of acute alcohol intoxicated liver sections was recovered by APS with just mild steatosis and necrosis [31]. Administration of MP-1 alleviated liver damage due to attenuated alcohol-induced oxidative damage [30].

The antioxidant enzymes SOD, GSH-Px, and CAT are an important defense system against oxidant stress. Improved antioxidant enzyme activities along with decreased MDA levels by PHGG contributed to the protective effects of PHGG against acute alcohol-induced liver injury (Table 2). A water-soluble polysaccharide isolated from *Crassostrea gigas* (CGPS-1) was found to significantly decrease MDA levels while increasing SOD activity in acute liver injured mice [15]. SOD, CAT, and GSH-Px activities in alcohol injured liver tissues were restored and MDA content was attenuated after treatment with intracellular mycelium polysaccharides from *Pleurotus geesteranus* [32]. Analogously, administration of an acidic polysaccharide from *Schisandra chinensis* along with alcohol significantly increased the activities of SOD and MDA when compared in rats with those that consumed alcohol alone [33]. 

Alcohol-induced oxidative stress can interfere with lipid homeostasis. When exposed to alcohol, the effect of the AMPK signaling pathway on preventing steatosis might be weakened and steatosis could be aggravated by ROS [34]. Thus, AMPK provides an especially promising pharmacological target for acute or chronic alcohol-induced lipid accumulation [10]. Reduced AMPK also causes a change in its downstream PPARα and SREBP1, which are widely expressed in adipose liver [34,35]. It was reported that up-regulated hepatic expressions of PPARα genes and down-regulated SREBP1 genes were thought to be responsible for acute alcohol-induced dyslipidemia [36,37]. SREBP1 is the major transcription factor regulating relative genes of hepatic fatty acid and TG synthesis, and can up-regulate cytochrome p4502E1 (CYP2E1) expression via free fatty acids, and further control hepatic lipid oxidative stress and lipid peroxidation [37,38]. Meanwhile, lipogenesis in the liver tissue can be stimulated by alcohol treatment through the activation of FASN [38]. This results in lipid accumulation and thus aggravation of the hepatotoxicity caused by acute ethanol exposure. In this study, co-administration of PHGG recovered the phosphorylation of AMPK and downstream ACC protein, increased PPARα expression, and suppressed FASN expression and activation of SREBP1 (Figure 3). Similar results have been described for extracts of root bark of *Ulmus davidiana var. japonica* and *Pleurotus citrinopileatus* [10,34]. Dyslipidemia can be induced by acute alcohol exposure in mice, such as the elevation of serum LDL-C level and hepatic lipid accumulation [32,39]. Although acute alcohol consumption could disorder blood lipids in healthy adults, the level of LDL-C, as a sign of dyslipidemia, has been indeterminate in different surveys [40,41].

Considerable evidence has suggested that both acute and chronic alcohol consumption can cause the translocation of Gram-negative gut bacteria and secretion of endotoxins, triggering activation of TLR-4-dependent signaling cascades in the liver and the induction of proinflammatory cytokines [42,43]. In the present study, endotoxins produced after alcohol consumption can bind to the TLR4 complex and activate inflammation via a signaling cascade including MyD88 and IκBα (Figure 4). PHGG administration down-regulated expression of inflammation related genes and suppressed the release of cytokines (Figure 4). Similarly, quercetin exhibited anti-inflammatory effects via preventing ROS-mediated activation of pro-inflammatory mediators and inhibiting NLRP3 inflammasome activation [44]. Fucoidan from *Fucus vesiculosus* reduced the production of inflammation-promoting cyclooygenase-2 and nitric oxide, while markedly increasing the expression of the hepatoprotective enzyme, hemeoxygenase-1, in murine liver and HepG2 cells [14]. Furthermore, findings of recent studies provide evidence that the gut microbiota play an important role in alcohol-induced liver injury, apparently through dysbiosis of the intestinal microbial ecosystem caused by binge drinking [45,46]. Treatment with a high-fiber diet can counteract hepatocyte pathology and gut leakage, and thus could be a promising therapeutic option [45,46]. PHGG, a dietary fiber that is readily fermentable, can stimulate proliferation of beneficial Bacteroidetes (Bacteroides and Parabacteroides) with positive health markers and outcomes [27,47].

CYP2E1 is responsible for alcohol metabolism, producing ROS of superoxide radical, hydrogen peroxide, nitric oxide, and hydroxyl radical [3]. ROS induced by alcohol can activate apoptosis, a prominent feature of ALD at preceding stages [5,9]. Caspases play an essential role in programmed cell death, with capase-3 the key enzyme in the execution phase of cell apoptosis [2,4]. Inhibitors of caspases are effective to prevent apoptosis, and thus can ameliorate liver injury in animal models from acute liver diseases [1]. Meanwhile, when experiencing stress and/or DNA damage, PARP can be activated for protection [48]. The TUNEL assay indicated substantial increase of apoptosis in the liver tissues of ALD mice (Figure 5A,B). PHGG administration attenuated alcohol-induced liver cell apoptosis with suppressed cleavage of caspase 3 and PARP (Figure 5C). Meanwhile, CYP2E1 expression in liver tissues was effectively suppressed by PHGG, and thus attenuated alcohol-induced oxidative stress (Figure 5C). *Antrodia cinnamomea* reduced the expression of cleaved caspase-3, -8, and -9 and the levels of phosphor-protein kinase B (Akt) in the alcohol-induced acute liver injury [1]. The mitochondria-dependent pathway is critical for the activation of caspases, and is mediated via Bcl-2 family proteins. Bcl-2 is an anti-apoptosis regulator to keep apoptogenic factors sequestered in the mitochondria. In contrast, Bax can induce the release of apoptogenic factors, and thus can be pro-apoptotic [49]. Alcohol exposure increased Bax expression but inhibited the expression of Bcl-2. PHGG supplementation decreased expression of alcohol-induced proapoptotic protein Bax, and prominently decreased the ratio of Bax/Bcl-2 (Figure 5E,F). In short, PHGG inhibited mitochondrial-mediated and caspase-dependent apoptosis through antioxidant activity, maintaining mitochondrial membrane integrity, and controlling apoptotic molecules’ release.

## 5. Conclusions

PHGG increased antioxidant enzyme activities, prevented alcohol-induced peroxidation on cell membrane lipids, and thus maintained membrane fluidity and integrity. Acute alcohol-induced metabolic imbalance of fatty acids in liver tissues was mediated through PHGG. PHGG administration down-regulated expression of inflammation related genes and suppressed the release of cytokines. Furthermore, both mitochondrial-mediated and caspase-dependent liver cell apoptosis were attenuated by PHGG. Thus, different metabolic pathways, such as antioxidant activities, lipid metabolism mediation, suppression of inflammatory mediator expression, and/or anti-apoptosis activities, contributed to the hepatoprotective effects of PHGG. Elucidation of the molecular mechanism will expand the utilization of PHGG as a functional food material for the treatment of alcohol-induced acute liver diseases.

## Figures and Tables

**Table 1:** Primer sequences used for RT-qPCR.

Genes	Primer Sequences (5′−3′)
PPARα	Forward: CTGAGGAAGCCATTCTGCGACATC
	Reverse: GCGTCTGACTCGGTCTTCTTGATG
SREBP1	Forward: AAGCAAATCACTGAAGGACCTGG
	Reverse: AAAGACAAGGGGCTACTCTGGGAG
TLR-4	Forward: CTGTATTCCCTCAGCACTCTTGATT
	Reverse: TGCTTCTGTTCCTTGACCCACT
MyD88	Forward: ATGGTGGTGGTTGTTTCTGACG
	Reverse: GTCGCATATAGTGATGAACCGCA
IκBα	Forward: AATCCTGACCTGGTTTCGCTCTT
	Reverse: ATCCTCGCTCTCGGGTAGCAT
NOS2	Forward: GGAGCGAGTTGTGGATTG
	Reverse: CCAGGCAGTAGGTGAGGG
GAPDH	Forward: TGGAGAAACCTGCCAAGTATGA
	Reverse: TGGAAGAATGGGAGTTGCTGT

**Table 2:** Effect of partially hydrolyzed guar gum (PHGG) treatment on lipid peroxidation and antioxidant status in liver.

Group	SOD (U/mg protein)	CAT (U/mg protein)	GSH-Px (U/mg protein)	MDA (nmol/mg protein)
Control	245.8 ± 15.5	34.0 ± 3.0	269.9 ± 10.1	1.56 ± 0.18
Alcohol	154.2 ±10.1 ^##^	21.4 ± 1.8 ^##^	143.8 ± 10.1 ^##^	6.64 ± 2.56 ^##^
Bifendate + Alcohol	194.4 ± 11.1 **	27.6 ± 2.6 *	215.7 ± 17.4 **	2.63 ± 1.11 **
PHGG-L + Alcohol	163.1 ± 14.3	27.7 ± 2.3 *	186.2 ± 11.0 **	4.43 ± 2.60
PHGG-M + Alcohol	198.8 ± 10.2 **	34.7 ± 1.9 **	236.1 ± 5.8 **	1.57 ± 0.50 **
PHGG-H + Alcohol	225.6 ± 10.4 **	43.9 ± 2.9 **	336.7 ± 17.3 **	2.26

Activities of antioxidant enzymes of superoxide dismutase (SOD), catalase (CAT), and glutathione peroxidase (GSH-Px), in liver were measured and used as indicators of antioxidant status at the end of experimental treatment. Malondialdehyde (MDA) level in liver was determined and used as an indicator of lipid peroxidation. Results were represented as mean ± SD (*n* = 10) of three independent measurements. “##” (*p* < 0.01), significantly different when compared to the Control group. “*” (*p* < 0.05) and “**” (*p* < 0.01), significantly different when compared to the Alcohol group. PHGG-L + Alcohol: PHGG low dose + alcohol group, treated with 500 mg/kg∙day of PHGG plus alcohol; PHGG-M + Alcohol: PHGG medium dose + alcohol group, treated with 1000 mg/kg∙day of PHGG plus alcohol; PHGG-H + Alcohol: PHGG high dose + alcohol group, treated with 2000 mg/kg∙day of PHGG plus alcohol.

**Table 3:** Effect of partially hydrolyzed guar gum (PHGG) treatments on serum lipid levels and adiponectin levels.

Group	TC (mmol/L)	TG (mmol/L)	LDL-C (mmol/L)	FFA (mmol/L)	ADPN (mg/L)
Control	2.62 ± 0.42	2.05 ± 0.04	0.63 ± 0.10	0.27 ± 0.01	13.6 ± 2.2
Alcohol	4.01 ± 0.24 ^##^	2.37 ± 0.13 ^##^	0.95 ± 0.04 ^##^	0.48 ± 0.05 ^##^	6.99 ± 0.86 ^##^
Bifendate + Alcohol	2.60 ± 0.27 **	2.03 ± 0.13 **	0.60 ± 0.06 **	0.31 ± 0.05 **	11.3 ± 1.4 **
PHGG-L + Alcohol	2.78 ± 0.43 **	2.10 ± 0.04 **	0.72 ± 0.08 **	0.37 ± 0.06 **	8.75 ± 0.86
PHGG-M + Alcohol	2.69 ± 0.33 **	2.11 ± 0.07 **	0.67 ± 0.08 **	0.31 ± 0.05 **	10.6 ± 1.4 **
PHGG-H + Alcohol	2.53 ± 0.21 **	2.09 ± 0.08 **	0.69 ± 0.04 **	0.27 ± 0.03 **	9.15 ± 1.20

Fasting serum levels were measured after mice were fasted for 12 h at the end of four weeks’ treatment. Serum levels of total cholesterol (TC), triglyceride (TG), low density lipoprotein-cholesterol (LDL-C), free fatty acids (FFA), and adiponectin (ADPN) were measured and used as indicators of lipid metabolism. Results were represented as mean ± SD (*n* = 10) of three independent measurements. “##” (*p* < 0.01), significantly different when compared to the Control group. “**” (*p* < 0.01), significantly different when compared to the Alcohol group. PHGG-L + Alcohol: PHGG low dose + alcohol group, treated with 500 mg/kg∙day of PHGG plus alcohol; PHGG-M + Alcohol: PHGG medium dose + alcohol group, treated with 1000 mg/kg∙day of PHGG plus alcohol; PHGG-H + Alcohol: PHGG high dose + alcohol group, treated with 2000 mg/kg∙day of PHGG plus alcohol.
